# Role of the microenvironment in myeloid malignancies

**DOI:** 10.1007/s00018-017-2725-4

**Published:** 2017-12-08

**Authors:** Marie Goulard, Christine Dosquet, Dominique Bonnet

**Affiliations:** 1 INSERM, UMRS1131-Paris Diderot University, Saint Louis Hospital, Paris, France; 2 Cell Biology Department, APHP, Saint Louis Hospital, Paris, France; 3 Haematopoietic Stem Cell Laboratory, The Francis Crick Institute, 1, Midland Road, London, NW1 1AT UK

**Keywords:** Bone marrow niche, Myeloid malignancies, Leukemic-initiating cells, Mesenchymal stroma cells

## Abstract

The bone marrow microenvironment (BMM) regulates the fate of hematopoietic stem cells (HSCs) in homeostatic and pathologic conditions. In myeloid malignancies, new insights into the role of the BMM and its cellular and molecular actors in the progression of the diseases have started to emerge. In this review, we will focus on describing the major players of the HSC niche and the role of the altered niche function in myeloid malignancies, more specifically focusing on the mesenchymal stroma cell compartment.

## Introduction

The HSCs, the cell population capable of reconstituting the entire blood system, have been highlighted for the first time by Till and McCulloch [1, 2]. Several functional techniques both in vitro and in vivo are able to demonstrate the presence of the HSCs. These techniques are indirect, proving the presence of the stem cells by their capacity of differentiation, i.e., in vitro, the colony forming cells (CFC) and the long-term culture-initiating cells (LTC-IC) assays [3]. The best proof of the presence of HSCs is the xenograft experiment: when injected in sub-lethally immunodeficient mice, the HSCs are capable of long-term reconstitution of the entire hematopoietic system and capable of repopulation into secondary recipient mice, thus proving their stem cell multi-lineage and self-renewal status [4].

The HSCs are located in BM “niches”. The concept of stem cell niches has been introduced for the first time by Schofield [5]. By studying CFU-S, he stated that the HSCs needed to be associated with other cells to determine their behavior. The niche concept is a way of explaining the dependence of HSCs to their microenvironment [6]. This concept implies that HSCs would not have the same self-renewing capacity without the support of the BMM cells. Niches are composed of cellular and molecular components that regulate the fate of the HSCs, i.e., quiescence, self-renewing capacities, differentiation and mobilization [7, 8]. More recently, the concept of the niche determining the fate of the HSC has been reexamined. Indeed, HSC fate has been proven to be highly specific, both in vivo and in vitro and defined by intrinsic epigenetic features [9]. The niche-restricted HSCs differentiation only to maintain their stem cell status, and thus having a permissive rather than instructive role [9]. Nevertheless, stroma modifications have been reported to contribute to abnormal hematopoiesis such as myelodysplastic syndromes (MDS), myeloproliferative neoplasms (MPN)-like disease (see paragraph: mouse models to study the role of BMM in myeloid malignancies). Thus, these studies represent strong evidence that the microenvironment exerts more than a mere by standard effect in myeloid malignancies. The relationship between the niche and the malignant clone(s) has reactivated the concept of the “bad seed in the bad soil” theory proposed in 1889, by Stephen Paget, an English surgeon, to describe how bad environment (“the soil”) of tumor cells (“the seeds”) could favor metastasis development [10, 11].

## Normal bone marrow microenvironment

**Fig. 1:**
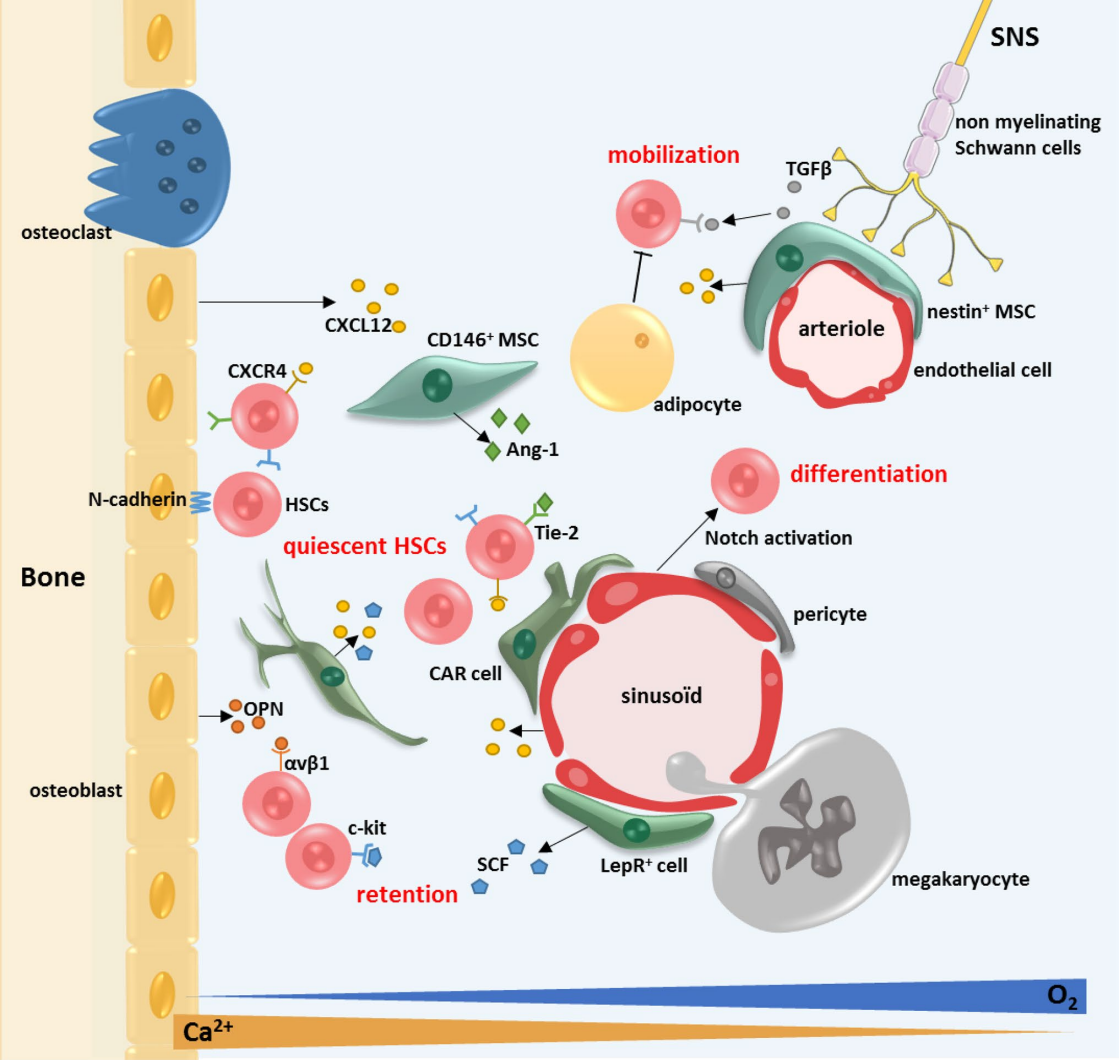
The normal bone marrow microenvironment. The HSCs’ fate is regulated by a specific microenvironment in the BM: the niches. The endosteal niche is commonly described close to the endosteum and is composed of osteoblasts and osteoclasts. The vascular niche is in the central zone of the bone, close to the sinusoids. Several types of cells compose these niches: the MSCs (LepR^+^ cells, CAR cells, nestin^+^ cells and CD146^+^ cells), the endothelial cells, the megakaryocytes, the adipocytes and the sympathetic nervous system (SNS). Several couples of molecules are implicated in the HSCs fate: particularly CXCL12–CXCR4, SCF—c-kit, angiopoietin 1 (Ang-1)—Tie-2, thrombopoietin (TPO)—MPL and osteopontin (OPN)—αvβ2 integrin

The bone marrow microenvironment (BMM) is a complex cellular and molecular entity composed of mesenchymal stroma cells (MSC), endothelial cells, nerves from the sympathetic nervous system, accessory cells (T lymphocytes and monocytes), etc. that plays a role in BM homeostasis (see Fig. 1). This cellular and molecular microenvironment regulates the HSCs quiescence, their self-renewal and their differentiation via cellular interactions and paracrine effects [7]. MSCs play a central role in the interactions with HSCs. Vascularization plays also a major role in the BM. There are two types of blood vessels: the fenestrated sinusoids in the endosteum and the medulla, and the arterioles in the endosteum. BMM is hypoxic displaying a decreasing gradient of oxygen between the BM sinusoids and the bone remodeling units (6–1%) [7, 8], due to the dense vascularity of the center-medullary zone of the BM. Hypoxia is inducing HIF-1α and hypoxia-inducible factor (HIF-2α), essential factors for the long-term self-renewing of the HSCs [12]. There is also a high concentration of calcium ion (Ca^2+^) in the endosteal region due to bone remodeling [13]. In this microenvironment, the cross-talk between MSCs and HSCs is imperative for the homeostasis of adult BM.

A number of cytokines, cytokine receptors and adhesion molecules have been implicated in the cross-talk between HSCs and cells of the BMM, particularly CXCL12 (SDF-1)—CXCR4, SCF (stem cell factor)—c-kit, vascular cell adherence molecule 1 (VCAM1)—VLA-4 (α4β2), angiopoietin-1 (Ang-1) and Tie-2, thrombopoietin (TPO)—MPL [14]. In vitro studies showed that the BMM cells, notably the osteoblasts secrete hematopoietic cytokines such as CXCL12, SCF, Ang-1 IL-6 and Jagged 1 (Jag1, Notch ligand) [15, 16]. SCF and IL-6 support normal hematopoiesis but also the maintenance and quiescence of HSCs. SCF is principally expressed by the perivascular cells in the BM [17]. Ang-1 regulates HSCs quiescence and has an anti-apoptotic effect [14]. TGFβ1 (transforming growth factor β1) has also been demonstrated to induce HSCs quiescence [18]. Osteopontin (OPN) is a glycoprotein synthesized by osteoblasts in the endosteal region that recognizes the integrin αvβ1 on the HSC surface and plays a role in cell adhesion, inflammatory responses and angiogenesis. OPN also plays a role in the localization, proliferation and mobilization of HSCs [19]. A mouse model of OPN deficient mice shows that OPN retains the HSCs in the BM and regulates negatively their number [20]. The osteoblasts express TPO at their surface that activates the cMPL receptor present at the surface of the hematopoietic stem and progenitor cells (HSPCs). This TPO/cMPL interaction induces the quiescence of the stem cells via activation of the β-integrin pathway [21].

### The endosteal and vascular niches

Usually two types of niches are described: the endosteal niche and the vascular niche but theses niches are tightly linked anatomically and functionally inside the trabecular bone [7, 22, 23]. The HSCs niches are dynamic structures responding to physiological demands. In general, the closer the HSCs are to the endosteum, the more quiescent they are [23–25], proliferative HSCs being mostly located in the central zone of the BM.

The endosteal niche is located closer to the bone surface and mainly plays a role in the quiescence of the HSCs. The main actors of the HSCs regulation in this niche are the osteoblasts and osteoblastic progenitors. The osteoblasts are distributed along the endosteal surface of the bone. They are in close proximity to the vessels [24, 25]. In a transgenic mouse model with PTH/PTHrP receptors activated in the osteoblasts, the increase number of these cells was associated with an increased number of HSCs, and HSCs cell growth in the endosteal region of the BM [15]. The osteoblast cells support the HSCs and influence their functions through Notch activation. Notch inhibition induces a decrease in the number of HSCs in the endosteal region [26]. The inactivation of the bone morphogenetic protein receptor (BMP) in another mouse model was shown to increase the number of osteoblasts and HSCs. The cell-to-cell contact between the HSCs and the osteoblasts is mostly mediated by N-cadherin and β-catenin [15, 27].

The vascular niche was first described as supporting the proliferation and differentiation of HSCs but there is now evidence of quiescent HSCs in the vicinity of sinusoids [17]. A study shows that nearly 85% of the long-term repopulating HSPCs are close to sinusoids [28]. In this zone, the HSCs are in contact with leptin receptor (Lepr^+^), CXCL12^high^ cells [28] and endothelial cells who promote their maintenance [17]. The HSCs are physically close to the sinusoids and distant from the arterioles [28]. The Lepr^+^ cells are surrounding the sinusoids. NG2^+^ cells, or polydendrocytes, are close to the arterioles in the endosteum, and play also a role in the maintenance of the HSPCs. The depletion of NG2^+^ cells induces HSCs proliferation and exhaustion of HSCs pool [29].

### The mesenchymal stromal cells (MSCs)

In 1867, Cohnheim described for the first time, particular non-hematopoietic cells in the bone marrow capable of regeneration. It is only in 1970, that Dr. Friedenstein described the isolation of (MSC) [30] which has been described to be in close proximity of the HSCs within the BM [31] and were later isolated using the Stro-1 antibody [32]. Nowadays, the mesenchymal stromal/stem cells are defined by the International Society of Cellular Therapy (ISCT) by their capacity of adhesion to plastic; their capacity to differentiate toward adipogenic, osteogenic and chondrogenic pathways and their specific phenotype [33]. The MSCs are described as a heterogeneous group of cells sharing the same positive and negative phenotype: CD73^+^, CD90^+^, CD105^+^ and CD34^−^, CD31^−^, CD45RA^−^, CD14^−^, CD19^−^, HLA-DR^−^. These criteria allow the standardization of MSCs characterization.

The MSCs are rare elements in the BM (0.01%) [34] but play a major role in the relationship between the BMM and the HSCs, in particular the CAR cells (CXCL12-abundant reticular cells), the nestin^+^ cells and the CD146^+^ cells [35]. The origin of the MSCs in the adult BM are the LepR^+^ (leptin receptor) cells. The LepR^+^ cells represent 0.3% of the cells in the BM and are quiescent but proliferate under stress [36].

The CD146^+^ cells are a subtype of MSCs mostly located in the human vascular niche. They represent 3% of the mononuclear cells inside the BM. The CD146^+^ cells express Ang-1 and CXCL12 and interact with HSCs and endothelial cells by their expression of Tie-2 and CXCR4 [37].

The nestin^+^ cells are mostly perivascular. They represent 0.08% of the mononuclear cells inside the BM and derive from the neural crest [37]. The nestin^+^ cells are also associated with nerves from the sympathetic nervous system (SNS) [35, 38]. This specific type of MSCs supports the homing and reduces the mobility of HSCs. The nestin^+^ cells also regulate the quiescence of HSCs via a high expression of maintenance genes: CXCL12, SCF, Ang1, IL-7, VCAM-1 and OPN [37]. In vitro, the nestin^+^ cells loose rapidly the nestin at their surface because of cell differentiation.

The CAR cells are more abundant than the nestin^+^ MSCs (0.27%). This type of MSCs is mostly located in the endosteal region of the BM. The CAR cells regulate the cell cycle and the self-renewing of the HSCs via a high expression of CXCL12 and SCF. It seems that most quiescent HSCs are in close contact with CAR cells [39]. This function has been confirmed in an in vivo model of CXCL12 silencing in the CAR cells [40]. In this model, the number of HSPCs is decreased. The CAR cells control the proliferation and the maintenance of the HSC pool [10, 41].

### Endothelial cells

MSCs and HSCs are in close contact with the endothelial cells in the BM. Several evidences point out the key role of the endothelial cells in the regulation of the HSCs in the BM niche. In a mouse model with disruption of VEGFR2 and VE-cadherin, a decreased number of HSPCs, with more differentiated cells was observed [42]. A deletion of E-selectin at the surface of endothelial cells promotes the quiescence of the HSPCs [43]. In this *Sele* ^−/−^ mouse model, BrdU assay shows reduced HSCs turnover—more than 30% of HSC in G_0_ phase—compared to wild type or P-selectin knockout mice. These studies suggest clearly that the endothelial cells participate to the proliferation and quiescence of the HSCs.

### Other cells, actors of the HSCs niche

Several other cell types play a major role in the regulation of the HSCs fate in the BMM such as the megakaryocytes, the adipocytes, nerves from the SNS and macrophages. The megakaryocytes are associated with the vascular niche, are in tight contact with HSCs and regulate their proliferation through various cytokines (IGFBP-3, IGF-1). They also regulate the HSCs quiescence by secreting platelet factor 4 (PF4—CXCL4) [44, 45]. In a mouse model, the absence of megakaryocytes leads to a loss of HSCs quiescence and enhances differentiation [45]. The role of megakaryocytes in the HSCs quiescence is also mediated by TGFβ1 contained in their α-granules together with PF4 [46].

The adipocytic tissue in the BM has a mesenchymal origin [47]. Several evidences point to the fact that the adipocytic cells support the HSCs maintenance and proliferation in their niche by producing the adipokine and adiponectin [48]. Interestingly, the number of adipocytes is inversely proportional to the number of HSCs and their decrease enhances the HSCs engraftment [49]. A recent study has shown that the adipocytic cells inhibit hematopoiesis and BM regeneration via the release of dipeptidyl peptidase 4 (DPP4 or CD26), a protein associated with apoptosis and immune response [50].

The sympathetic nervous system (SNS) is implicated in the mobilization of HSCs in the BM [51]. The BM neurons are β-adrenergic nerve terminals [51, 52]. The SNS is composed of non-myelinating Schwann cells that regulate the niche by activation of TGFβ1 [52]. The SNS regulates CXCL12 expression by perivascular BMM cells and HSCs retention via the circadian oscillation [53, 54].

The macrophages have been described as important elements of the BM niche. In a mouse model, the deletion of CD169^+^ cells (macrophages) is deleterious for the retention of the HSPCs by the niche [55]. The macrophages express also CXCL12 and osteocalcin [56] and are capable of modulating the CXCL12 expression by MSCs [55, 57] leading to the retention of the HSCs in the niche and to support the survival of osteoblasts. The use of granulocyte colony-stimulating factor (G-CSF) decreases the number of osteoblastic cells and depletes the macrophages in the endosteum [57], suggesting that macrophages support the osteoblasts in the retention of the HSCs.

## BMM in myeloid disorders

### Mouse models to study the effect of BMM in malignant development

Mouse models have been used to better understand the progression of myeloid malignancies and particularly the role of the BMM in their natural history. A mouse model of Osx-GFP-Cre^+^ Dicer 1^fl/fl^ mice suggests that a modification of the BMM could in itself induce MDS [58]. In this model, the deletion of Dicer1 gene in osteoprogenitors leads to an impaired hematopoiesis mimicking human MDS. In the osteoprogenitors of these mice, the expression of the Shwachman–Diamond–Bodian (SBDS) gene decreases. Moreover, KO SBDS mice display a MDS phenotype too. In a mouse model with a deletion of SBDS gene, the MSCs display non-functioning mitochondria associated with higher oxidative stress and DNA damage in the HSCs, suggesting a possible role of the stroma in the development of a myeloid disease [59].

In an acute myeloid leukemia (AML) mouse model, the overactivation of the β-catenin pathway via overexpression of a constitutive active mutation of β-catenin in osteoblasts reduces the differentiation potential of myeloid and lymphoid progenitors, leading to MDS/AML progression [60]. In these osteoblastic cells, overexpressing beta-catenin the authors revealed an upregulation of NOTCH ligand Jagged-1. They went on to demonstrate that in human AML BM biopsies, a nuclear-activated β-catenin in osteoblasts and elevated Notch signaling in hematopoietic cells providing evidence of the potential relevance of these results in human disease. Interestingly, the alteration of osteoblastic cells by activation of the PTH receptor in BCR-ABL induced mouse model of chronic myeloid leukemia (CML), decreases CML disease but enhances MLL-AF9 induced AML development, demonstrating that distinct myeloid disease required different niches factors [61].

Several other mouse models demonstrate the implication of the BMM in the development and progression of non-Philadelphia MPN. MPN disease was able to develop in a mouse model where deletion of the retinoblastoma (RB1) protein was introduced in both the hematopoietic compartment as well as the microenvironment [62]. In another study by the same group, a deletion of retinoic acid receptor gamma (RARγ) in the niche was sufficient to induce a MPN-like disease [63]. In another study, Ptpn11-activating mutation in the nestin^+^ MSCs but not in osteoblasts or endothelial cells, has a deleterious effect on HSCs and leads to MPN [64]. PTPN11-mutant MSCs overproduced CCL.3, which recruited monocytes secreted IL1β and other proinflammatory cytokines to the HSC niche.

The number of nestin^+^ cells in the BM and the expression of nestin messenger were reduced in the BM of Jak2^V617F^ mice, associated to expansion of leukemic initiating cells (LICs) and MPN progression [65]. In another murine model of MPN (*Scl*-*tTA:TRE*-*BCR/ABL* double transgenic mouse model), the influence of LICs on the microenvironment was associated with an increase in abnormal osteoblastic cells in the BM creating a myelofibrosis inflammatory environment. This “leukemic niche” promoted proliferation of LICs instead of normal HSCs, TGFβ and Notch pathways were implicated in this BM remodeling [66].

## BMM modifications in patients with myeloid malignancies

In acute and chronic myeloid malignancies, the cross-talk of the neoplastic myeloid cells with the BMM plays an important role in the progression of the disease. In patients with myeloid neoplasia, there are morphological modifications of the BMM such as an increase of angiogenesis in patients with AML and MDS [67–69]. Similar angiogenesis and impair vascularity was also observed in AML-PDX model [70]. BM fibrosis is frequently observed in patients with non-Philadelphia MPN [71] and in patients with MDS [72].

In patients with myeloid malignancies, a possibility to approach the modifications of the BMM is to isolate and study the BM MSCs. Indeed, a number of studies suggest that functional modifications of the BM MSCs are related to the natural history of myeloid diseases such as AML, MDS, non-Philadelphia MPN and CML [73, 74].

**Fig. 2:**
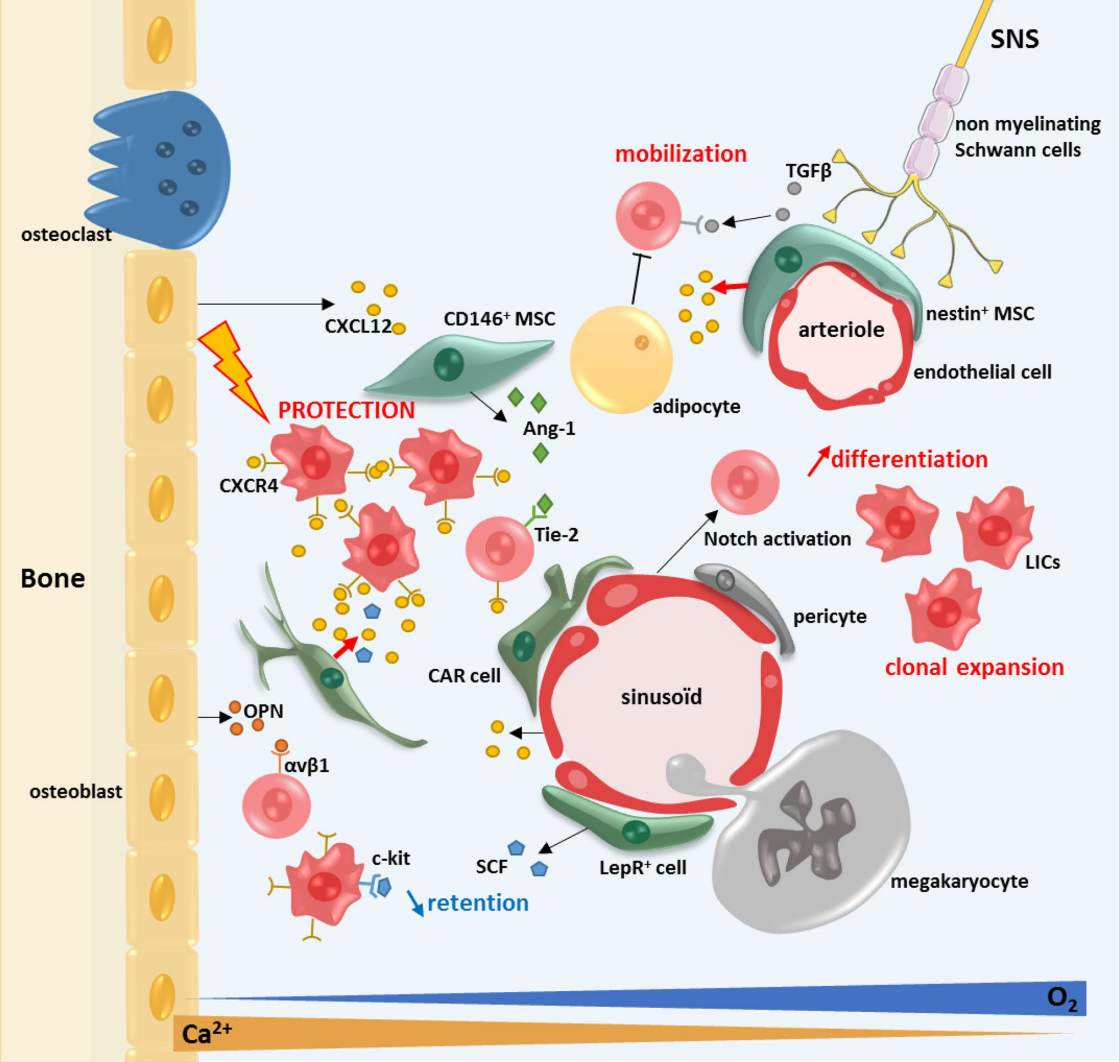
The bone marrow microenvironment in myeloid malignancies. The BMM confers a protective environment from apoptosis for the LICs via the CXCR4/CXCL12 axis. CXCR4 is highly expressed at the surface of LICs and CXCL12 is highly expressed by the MSCs. The maintenance and retention of the HSCs in the BM are decreased. The diminution of retention of the HSCs by the BMM is mediated by an impaired production of SCF by the MSCs

Here, we choose to focus on the genetic, epigenetic, gene expression, clonogenic and differentiation capacities of the MSCs of patients with myeloid neoplasia as well as bone marrow failure syndrome exemplified by Aplastic anemia (see Fig. 2).

### The BMM of aplastic anemia (AA)

Aplastic anemia is a BM failure, associated with a hypoplasia and peripheral pancytopenia. Changes in the BMM of AA patients have been reported. In BM biopsy from AA patients, an increase of stromal cells expressing osteopontin and a decrease of osteonectin expressing cells as well as endothelial cells have been described [75–77]. The AA BM has a decreased angiogenesis [77, 78] associated with a decrease of VEFG expression [79].

A number of studies have reported on AA MSCs, and showed that in general AA MSCs have either a normal or slightly decreased clonogenic potential compared to control [75, 80–82]. The AA MSCs are more incline to enter apoptosis in vitro [75]. Studies on MSCs differentiation from AA patients are heterogeneous and do not allow us to conclude [75, 77, 83].

One study reported that AA MSCs have a reduce capacity to support a normal hematopoiesis in vitro [83]. But in a 3D in vivo scaffold, AA MSCs were capable to form a functional BM niche [81].

Several genes involved in biological processes such as proliferation, chemotaxis and interaction with HSCs are downregulated in AA MSCs [74]. VCAM-1 plays a crucial role in HSCs retention in the BMM and is particularly decreased in AA MSCs [83, 84]. AA MSCs secrete high levels of macrophage inflammatory protein 1 alpha (MIP-1alpha) and GM-CSF but low levels of IL-1Ra compared to healthy control MSCs [85]. This abnormal gene expression in AA MSCs could explain at least partly the abnormal HSCs regulation observed in AA patients.

### The BMM of MDS

MDS constitute a heterogeneous group of clonal myeloid diseases with diverse phenotypes, characterized by ineffective hematopoiesis with varying risk of leukemic transformation. In vitro, MDS stromal cells were reported to be quantitatively and functionally impaired.

The results of cytogenetic analysis of MSCs from MDS patients are contradictory [86–89]. A study by Lopez-Villar reported no cytogenetic abnormalities in the MDS MSCs despite cytogenetic abnormalities in the HSCs [87]. Other studies reported abnormalities of karyotype in MSCs obtained from MDS patients [73, 88]. The corresponding HSCs also displayed abnormalities but none were similar to those observed in the corresponding MSCs. It is important to underline that MSCs are known to be genetically instable in culture [89]. MDS-MSCs have a different methylation profile than normal MSCs. An increase of the methylation in genes involved in processes linked to cellular phenotype and transcriptional regulation has been reported [90].

A large majority of these studies deals with ex vivo expanded MSCs. In cultured, MDS-MSCs modification of expression of various genes has been observed: such as cytokines [91–94], adhesion molecules [95] and molecules involved in the interaction with the HSCs such as OPN, Jagged1, Kit-L and Ang1 [90]. CXCL12 was reported to be overexpressed in MSCs of MDS patients [94, 96, 97].

A recent study of isolated mesenchymal elements sorted directly from low-risk MDS BM patients by the markers CD45^−^/7AAD^−^/CD235a^−^/CD31^−^/CD271^+^/CD105^+^ describes a different transcriptomic signature of MSCs from MDS patients in comparison with normal MSCs or cultured MDS-MSCs. The directly sorted uncultured MSCs have characteristics of cellular stress and upregulation of inflammation cytokines linked to inhibition of hematopoiesis [97]. Several studies demonstrated epigenetic and transcriptomic variations of cultured MDS MSCs. This was further confirmed in directly isolated mesenchymal elements compared to control MSCs with a specific increase in hypermethylation in enhancer regions [98, 99] and downregulation of pathways including the Wnt pathway [97, 100]. Interestingly, there were also differences both at the epigenetic and transcriptomic level, between the native sorted stromal elements and cultured MSCs clearly, indicating that 2D-cultured system used for expanded MSCs (normal or from patients) influence these cells [97, 100].

MDS-MSCs clonogenic potential was shown to be clearly decreased at diagnosis as demonstrated by CFU-F assays [88, 95, 97, 101, 102]. Similarly, the proliferative capacity of MDS-MSCs is also decreased, no matter the patient IPSS score [90, 95, 102, 103]. The MDS-MSCs enter senescence earlier than normal MSCs [104, 105] and display a senescent phenotype in vitro [97].

The MSCs differentiation data are heterogeneous. Some studies show no alterations of the differentiation capacity of the MDS MSCs [86, 91, 92, 101, 103, 106]. The adipogenic and chondrogenic pathways were reported as decreased in some studies [87, 107]. The osteogenic pathway is often reported as impaired in high-risk and low-risk MDS [102, 104, 108]. Genes linked to the osteoprogenitors and osteoblasts (Dicer, DROSHA, RUNX2 and SBDS) have been reported to have a weaker expression in the MDS-MSCs [58, 108, 109]. As suggested by a mouse model, the MSCs, especially those differentiating in the osteogenic pathway, could have a primary role in MDS development [58].

It has been demonstrated that MDS-MSCs have an impaired capacity to support a normal hematopoiesis in vitro [90, 94, 95, 102]. MDS-ICs have been reported to proliferate less when not co-cultured with their autologous MSCs [108]. When co-cultured with MDS-ICs, normal MSCs have a higher expression of LIF, VEGF, ANGPTL4 and CXCL12 than the non-co-cultured counterpart [96, 107].

In vivo, primary MDS cells engraft poorly in the immunodeficient mice [110]. A potential increase of MDS engraftment was reported when co-injected with either control or MDS MSCs in NSG-S mice (3/20 samples) [111]. Nevertheless, a recent study showed no difference of MDS engraftment in NSG or NSG-S mice when co-injected with MDS-MSCs [112]. Thus, the long-term effect of MDS-MSC on MDS engraftment is unclear, probably due to the fact that the implantation of MSCs (even after intra bone injection) in immunodeficient mice, do not really engraft long-term [112]. So far, there is no study using the 3D-scaffold model for MDS engraftment assessment in vivo.

### The BMM of chronic MPN

Myeloproliferative neoplasms are a group of chronic myeloid diseases characterized by a BM hyperplasia of one or several myeloid lineages. Among MPNs are CML with Philadelphia chromosome and bcr-abl transcript and the non-Philadelphia MPN, i.e., polycythemia vera (PV), essential thrombocythemia (ET) and primitive myelofibrosis (PMF). The most common mutations in non-Philadelphia MPN are Jak2^V617f^, MPL^W515^ and CALR mutations.

Few studies focus on the MPN-MSCs ex vivo. MSCs derived from the BM of MF patients display the same clonogenicity as normal MSCs in CFU-F assay and MF-MSCs have also been reported to have a similar capacity to support normal hematopoiesis as normal MSCs [113].

Nevertheless, the remodeling of BMM is particularly visible in the BM biopsies of pre-fibrotic MPN patients: the CD271^+^ cells are decreased in the endosteal and vascular niches and are associated with dysplastic megakaryocytes [114]. Interestingly, a reduction in the number of sympathetic nerve fibers associated with the MSCs was reported in the BM of MPN patients [65].

The differentiation capacities of the MPN-MSCs seem to be impaired. Indeed, a report show in MF-MSCs, an increase capacity to differentiate into osteoblasts in vitro and to mineralize subcutaneously compared to normal MSCs and even MSCs derived from PV and ET patients [113]. In this study, this capacity was associated with an upregulation of the expression of Runx2, Dlx5, OPN and IBSP and a deregulated transcriptomic signature related to osteogenic lineage in MF-MSCs.

MPN-MSCs have also been shown to have a protecting role on LICs via paracrine secretion of IL-6, FGF and CXCL10 [115]. Two studies on PV patients report that the protecting role of MSCs against JAK2 inhibitor leading to a decrease in apoptosis of JAK2(V617F) mutated cells [115, 116]. In PMF patients, Jak2^V617f^ overactivates the complex CXCL12/CXCR4 via activation of the PI3K pathway and increases hematopoietic cells chemotaxis [116]. PV-MSCs overproduce fibrogenic and inflammatory cytokines such as TGF-β1 and BMP-2, which stimulate the osteogenic differentiation pathway [113, 117]. CD9 (tetraspanin 29) expression, known to be implicated in the HSCs interaction with the stroma, is decreased at the surface of CD34^+^ cells in PMF patients, this decrease being inversely correlated with fibrosis [118].

In a study using a 3D in vitro culture model, MPN-MSCs have been reported to have a decrease capacity to support HSCs and ET-MSCs to secrete lower levels of G-CSF and IL-17 [114]. In this study, the authors also reported an increased in fibronectin deposition by the MPN-MSCs, and confirmed this by tissue microarray of pre-fibrotic MPN-BM biopsies.

There are very few studies concerning CML-MSCs and their role in the CML BMM. A majority of studies have been done with stromal cell lines. The CML stromal cells have been reported to have a protective role on HSCs in vitro by decreasing their apoptosis [119], this role being mediated via the CXCR4/CXCL12 axis [120]. One study reported the presence of the same genetic aberration, particularly the fusion gene BCR-ABL, to be present in both the neoplastic clone and endothelial cells, suggesting that this molecular event occurred in hemangioblasts [121].

### The BMM of AML

AML is a heterogeneous clonal disorder characterized by expansion of immature myeloid progenitors (blasts) in the BM and peripheral blood. Chromosomic aberrations are frequently observed in MSCs of untreated AML patients. The most frequent are translocations [73]. These aberrations are never identical to those found in the HSCs of the same patients. Sequencing DNA from AML-MSCs also highlighted numerous gene mutations including mutations in plectin and chromatin remodeling genes [122]. Modifications of DNA methylation were also described: hypermethylation of PITX2 and HOXB6 genes and hypomethylation of HOXA3 and HOXA5 genes [123].

Gene expression modifications were also reported for a lot of genes including adhesion molecules [122], inflammatory cytokines [124–126], Notch pathway [127] and CXCL12 [128–130]. The link between MSCs and HSCs is mediated by CXCL12/CXCR4 in normal and in pathological conditions, particularly myeloid neoplasms [116, 120, 130]. CXCR4 is highly expressed on both normal and AML hematopoietic cells. A high level of CXCR4 expression on AML blasts is associated with a poor prognosis. In xenotransplantation models, the disruption of the interaction between CXCR4 and CXCL12 can induces an increase in the apoptosis rate and promotes leukemia regression, suggesting the disruption of the microenvironment protection [120, 130]. Inhibition of the expression of CXCR4 using an antagonist leads to AML blasts death by apoptosis. This cell death was mediated by the upregulation of miR-15a/miR16-1 which downregulates BCL-2, MCL-1 and cyclin-D1 [131, 132]. CXCL12 deletion in CAR cells and perivascular MSCs in genetically modified mouse model (*Cxcl12* ^*fl*^) decreases HSCs quiescence and self-renewal potential while CXCL12 deletion in osteoprogenitors enhances HSCs mobilization but does not affect their maintenance and quiescence [133, 134]. Furthermore, CXCL12 mRNA was reported to be more expressed by normal BM MSCs after co-culture with leukemic cells [96, 135].

In several studies, AML-MSCs clonogenic potential (CFU-F) was reported to be decreased at diagnosis [123, 125, 135]. Moreover, a study indicates that the CFU-F frequency was restored to a normal capacity when the patients were in complete remission [135]. In vitro, the AML BM MSCs proliferate less than normal BM MSCs and enter senescence faster [135]. Their maintenance in culture was shortened and may not exceed passage 2. On the contrary, another study using a large number of AML patients from good, intermediate and poor risk groups, indicated that AML-MSCs had an increase clonogenic potential and immunosuppressive capacity than healthy MSCs as well as an increase in anti-inflammatory signals like IL.10 [136].

The data on AML-MSCs differentiation are heterogeneous. Some studies indicate no alteration of this differentiation capacity [123–125, 135]. Other studies report a decrease of the osteogenic differentiation pathway, this decrease being correlated with a decrease expression of genes related to osteoblastic lineage such as osterix and osteocalcin [135]. A larger number of CD146^−^166^+^ osteoblastic cells were found in sections of BM of AML patients in comparison with normal BM [135]. Only in poor risk AML, Diaz de la Guardia et al., reported a decrease in adipo/osteogenic potential [136]. In two recent studies, AML-MSCs were reported to have a decrease in adipogenic and a parallel increase in osteo-lineage differentiation [137, 138]. In one of this study, they confirmed the decrease in adipogenesis in primary AML patients bone marrow and demonstrate that leukemic cells suppression of BM adipocytes led to an imbalance regulation of endogenous hematopoietic stem and progenitor cells, resulting in impaired myelo-erythroid maturation [137]. In the second study, they show that AML-induced osteo-lineage differentiation support leukemic growth [138].

The MSCs ability to sustain hematopoiesis can be studied both in vitro, via co-culture models in 2D and 3D and in vivo via xenografts in immunodeficient mice. AML-MSCs LTC-IC’s data suggest an impaired capacity of these cells to support a normal hematopoiesis [124–126, 135]. Few myeloid colonies are observed in methylcellulose after LTC-IC culture of normal HSCs with AML-MSCs. In addition, MSCs derived from AML patients were reported to support LICs in vitro better than healthy MSCs [136, 139]. Very few xenograft experiments involve the co-injection of AML cells together with autologous MSCs, but when co-injected, the MSCs do not seems to improve the level of engraftment [140]. Subcutaneous scaffold injected with MSCs in vivo using matrigel, or other materials, have recently been described and shown to improve the rate of LICs grafted in mice [141–143]. Similarly, implantation of these scaffolds, seeded with healthy MSCs, improve the engraftment of normal HSCs [141, 142]. The engraftment in the scaffold is reduced if the MSCs used are knockout for Hif-1α [140]. Thus, these 3D scaffolds seem to mimic the human niche environment and improve engraftment of the leukemic cells [140–143].

Several data suggest that the BMM has a protective effect on the leukemic cells. The contact with MSCs protects the leukemic cells from apoptosis [127, 144] and from various chemotherapies [127, 145–147]. It also maintains their undifferentiated status [139], self-renewing capacities [148] and survival [127, 144]. In vitro, AML-MSCs displayed the same chemoprotection capacities than healthy MSCs but have higher immunosuppressive and anti-inflammatory properties with a diminution of pro-inflammatory cytokines expression [136]. The protective effect of MSCs is modulated by various cytokines including the axis CXCR4/CXCL12 [127, 132, 145, 146]. On the other hand, AML cells influence the microenvironment and decrease the proliferation of normal MSCs in co-culture [123]. This observation suggests a bi-directional cross-talk between the LICs and the BMM in patients with AML.

## Discussion

Over the past decade, the contribution of different stroma cells to the maintenance of HSCs has started to emerge. A better understanding of the relationship between the HSCs/LICs and their microenvironment is a key to understand the natural history of myeloid neoplasia. Even though AA is a BM failure and not a myeloid malignancy, complication in AA can lead to secondary MDS or AML [149]. Thus, evaluating qualitatively and quantitatively the composition of the bone marrow stroma during the evolution of AA to MDS and AML and dissecting the differences at different malignant stages will be highly valuable to shed light into the specific role of certain microenvironment factors in myeloid malignancies and should help adapt therapeutics treatment targeting specifically the specific cross-talk between the malignant cells and their niche [150]. The difference in composition of the BM niche could also be possibly exploited as a potential clinical biomarker tool to predict response or relapse in clinical trial settings.

Drugs that disrupt adhesion of LICs from their protective niche have started to emerge in clinics such as CXCR4 and adhesion molecule (VCAM-1, VLA-4, E-selectin) inhibitors [150, 151 and see more details review 152]. As immunotherapy against MDS/AML are also expanding especially using different immune checkpoint inhibitors, the potential contribution of the stroma to immune escape to therapy resistance will have to be studied. Indeed, recently in pancreatic cancer, the CXCR4/CXCL12 axis has been linked to the resistance to immune checkpoint therapy [153]. Our understanding of the cellular composition of the normal, dysplastic, and leukemic niches and the complex interactions between these cells is still in its infancy. Nevertheless, targeting the deregulated niche and restoring niche function is already providing a promising new therapeutic rationale in myeloid malignancies.
